# Morphologies, Young’s Modulus and Resistivity of High Aspect Ratio Tungsten Nanowires

**DOI:** 10.3390/ma13173749

**Published:** 2020-08-25

**Authors:** Jianjun Gao, Jian Luo, Haibin Geng, Kai Cui, Zhilong Zhao, Lin Liu

**Affiliations:** 1 School of Mechanical Engineering and Automation, Fuzhou University, Fu Zhou 350108, China; gjj410zd@fzu.edu.cn (J.G.); genghb@fzu.edu.cn (H.G.); 2 School of Mechanical Engineering, Northwestern Polytechnical University, Xi’an 710072, China; ck2016@mail.nwpu.edu.cn (K.C.); zhaolong@nwpu.edu.cn (Z.Z.); 3 State Key Laboratory of Solidification Processing, Northwestern Polytechnical University, Xi’an 710072, China; linliu@nwpu.edu.cn

**Keywords:** tungsten nanowires, young’s modulus, resistivity, selective etching

## Abstract

High aspect ratio tungsten nanowires have been prepared by selective dissolution of Nickel-aluminum-tungsten (NiAl−W) alloys which were directionally solidified at growth rates varying from 2 to 25 μm/s with a temperature gradient of 300 K·cm^−1^. Young’s modulus and electrical resistivity of tungsten nanowires were measured by metallic mask template method. The results show that the tungsten nanowires with uniform diameter and high aspect ratio are well aligned. The length of tungsten nanowires increases with prolongation of etching time, and their length reaches 300 μm at 14 h. Young’s modulus of tungsten nanowires is estimated by Hertz and Sneddon models. The Sneddon model is proper for estimating the Young’s modulus, and the value of calculating Young’s modulus are 260–460 GPa which approach the value of bulk tungsten. The resistivity of tungsten nanowires is measured and fitted with Fuchs−Sondheimer (FS) + Mayadas−Shatzkes (MS) model. The fitting results show that the specific resistivity of W nanowires is a litter bigger than the bulk W, and its value decreases with decreasing diameter.

## 1. Introduction

Metallic nanowires have wide applications in various fields, such as micro-nano electronics, biotechnology, solar cells, sensors, and catalysis [1,2,3,4,5]. Metallic nanowires with small size and high aspect ratio are ideal materials for micro-nano devices, such as Zn, Ag, Ni, Au, and Cu micro-nanometer wires have been widely applied [6,7,8,9,10]. In order to realize the excellent properties of metallic nanowires, research on the mechanical and electrical conductivity of single metallic nanowire are fundamental and necessary.

The constitute phase in directionally solidified eutectic alloys has different dissolve potential. After selective etching the matrix phase, the single crystalline high aspect ratio metallic nanowires are produced [11,12,13,14,15]. Tungsten is a brittle metal, but it shows a surprisingly high flexibility on a nano-scopic scale [16]. Many works have focused on the morphologies of W nanowires by controlling the growth parameters and the dissolution conditions [17,18]. However, the effect of growth rate and etching time on the morphologies of W nanowires are seldom studied. While the growth rate and etching time affect the morphologies and structures of W nanowires, and thus may affect their mechanical properties and electrical transport properties. The electrical transport properties of metallic nanowires depend on its diameter [19]. To date, the resistivity of the single crystalline W nanowires in different diameter is unclear. Though Cimalla’s work has demonstrated that the Young’s modulus of W nanowires is close to the value of bulk W [16] and Hu proposed that the Young’s modulus graphene-coated tungsten nanowires shows a strong size effect [20], how the diameter affects the Young’s modulus of W nanowires is unknown. When testing the properties of a single nanowire, it needs to be assembled on a target table, and this usually is implement by focused ion beam (FIB) [16], photoetching methods [21], or nano-manipulation device [22], whose processes are time-consuming and costly. Therefore, a simple process to assemble the metallic nanowires on the target table is necessary.

In this work, the morphologies of tungsten nanowires with different diameters are investigated. W nanowires are assembled on a target by a metallic mask template method for test their Young’s modulus and resistivity. Young’s modulus of tungsten nanowires in different diameter is calculated by Hertz and Sneddon models. Resistivity of tungsten nanowires has been measured, and the surface and grain boundary effects on resistivity have been studied by combination of FS and MS models. This study provides an effective pathway for fabricating tungsten nanowires with excellent electrical conductivity and mechanical properties.

## 2. Experiment

**Figure 1:**
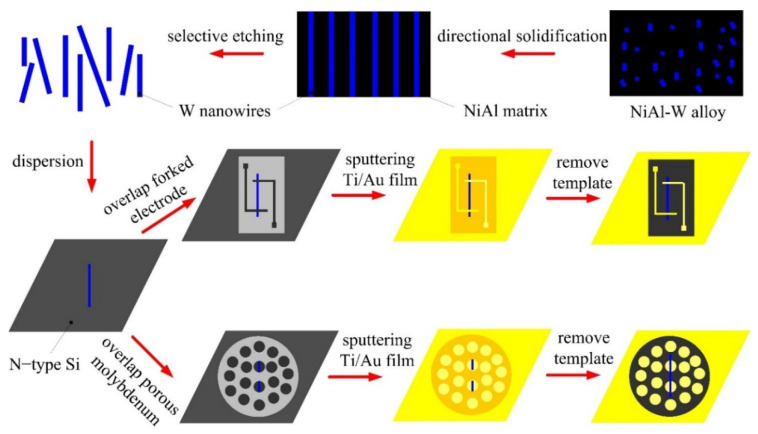
Schematic diagram of assembling a single W wire on the n-type Si by metallic mask template method.

Figure 1 illustrates the schematic diagram of assembling a single W wire on the N-type Si by metallic template method. The preparing W nanowires is synthesized of directional solidification and selective etching. First, NiAl–W eutectic alloy is directionally solidified at different growth rates with a temperature gradient of 300 K/cm [23]. NiAl matrix is selectively removed in 1 M phosphoric acid (H_3_PO_4_) solution at a constant potential of 3 V which is applied between working and counter electrode in a three-compartment cell. Second, the W wires are dispersed on surface of n-type Si. The forked electrode mask or porous molybdenum mesh is overlapped on surface of N-type Si. After that, composite metallic film (100 nm Ti/200 nm Au) are sputtered on the devices by magnetron sputtering. Last, the templates are removed, and a single W wire is assembled on n-type Si. By using metal mask template, W wire is assembled on the surface of n-type Si to test its elastic modulus and electrical transport properties.

An electrochemical workstation (CorrTest CS350, Wuhan Corretest Instruments Corp., Ltd, Wuhan, China) is employed in the electrochemical process. The morphology of W nanowires is observed by using a field emission scanning electron microscope (FESEM; Quanta 600FEG, FET, Hillsboro, OR, USA). An atomic force microscopy (AFM, Dimension FastScan, Bruker, Billerica, MA, USA) is used to measure the elastic modulus of W wires. Temperature and humidity test platform (Elite Tech Co., Ltd. AES−4^TH^, Beijing, China) is used to measure the electrical transport properties of single metal W wire.

## 3. Results and Discussion

### 3.1. Morphologies of W Nanowires

**Figure 2:**
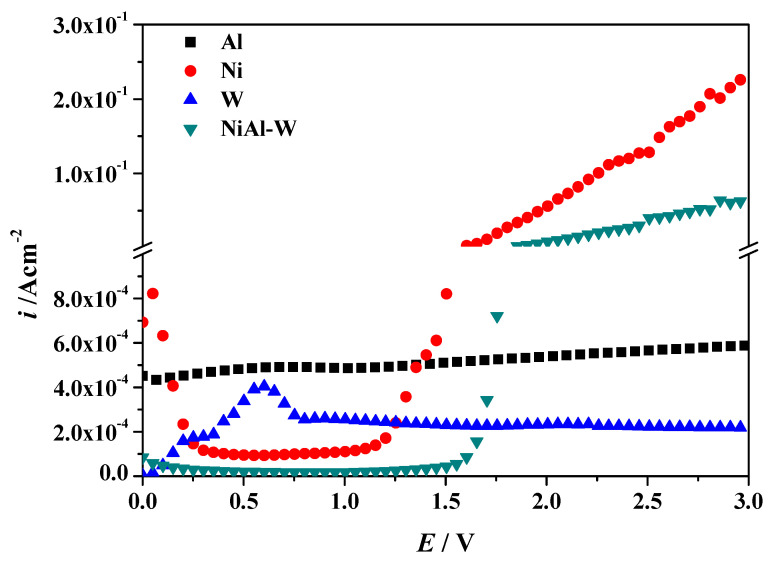
Potentiodynamic polarization curves of pure Al, Ni, W, and NiAl−W eutectic alloys in 1 M H_3_PO_4_. solutions, the sweep speed was 5 mV/s.

For selecting a proper anodization potential to etch NiAl matrix, potentiodynamic polarization curves of pure Al, Ni, W, and NiAl−W eutectic alloys in 1 M H_3_PO_4_ solutions are carried out (Figure 2). With the increase of corrosion potential, the current density of W increases first and then decreases, and its *E*−*i* curve has a peak value at 0.59 V. However, when the potential exceeds 0.76 V, the current density tends to be stable with the increase of corrosion potential. When at a potential of 3 V, W is passivated and free from etching. The current density of elemental Al is almost constant with the increase of corrosion potential, which indicates that the Al is passivated in phosphoric acid solution. The dynamic potential polarization curve of Ni is a typical passivation curve of metal anode. When the corrosion potential is between 0 and 0.25 V, Ni is at the initial passivation stage. When the corrosion potential increases to 0.5–1.2 V, Ni is in a stable passivation. At this stage, the current density no longer increases with the increase of the applied potential. However, when the corrosion potential exceeds 1.2 V, the current density increases significantly with the increase of corrosion potential, and Ni is in the trans-passive stage. While the potential exceeds 1.6 V, the current density increases sharply with the increase of corrosion potential. Therefore, when the corrosion potential is at 3 V, the Ni is corroded. When the applied potential is less than 1.6 V, the NiAl−W alloy keeps passivation. While the potential is more than 1.6 V, the current density increases sharply with the increase of applied potential. Therefore, when at 3 V, Ni element is corroded, W and Al elements are passivated. When Ni is etched, the NiAl matrix are removed and W nanowires are prepared [12].

**Figure 3:**
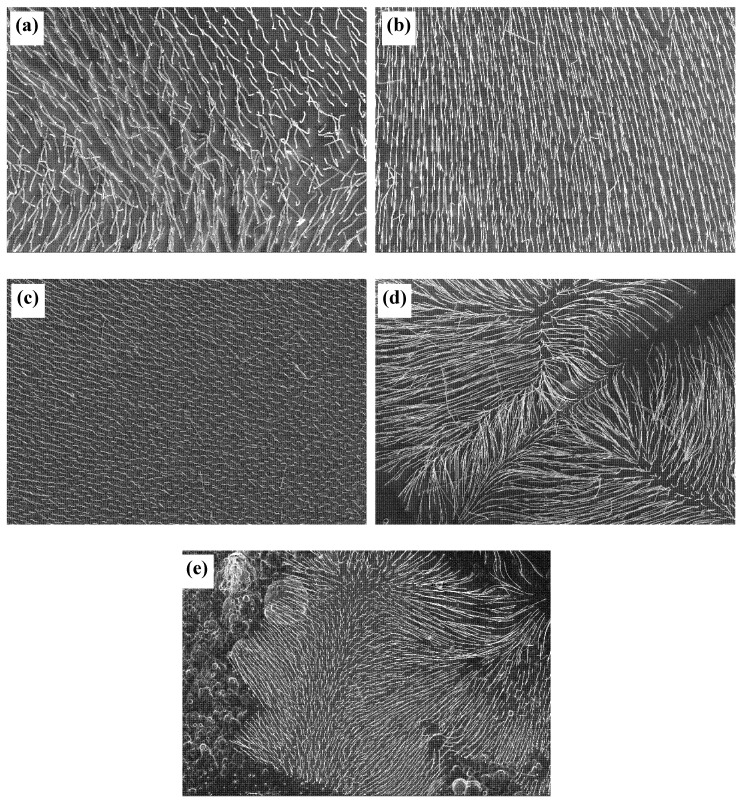
Morphologies of W fibers grown at different growth rates: (**a**) 2 μm/s, (**b**) 4 μm/s, (**c**) 6 μm/s, (**d**) 8 μm/s, and (**e**) 15 μm/s. The anodization time was 1 h.

Morphologies of W wires grown in different growth rates are illustrated in Figure 3. When the directionally solidified alloys are at a low growth rate, the solid–liquid interface is planar. Increasing growth rate leads to the cellular growth [23], and the cross-section morphology of W wires changes from hexagon to ellipse [18]. As shown in Figure 3, when the growth rate is 2–6 μm/s, W wires with a length of dozens of microns are uniformly distributed on the NiAl matrix. Due to the scraping and pushing action of hydrogen bubbles generated in the corrosion process, W wires are symmetrical length and uniformly arranged in a direction. When the growth rate is 8–15 μm/s, W wires diffuse from the center to the grain boundary. When etched at the same anodization time, the length of W wires at different growth rates are different. The length of W wires gained at 2 μm/s is about 20 μm, while its length decreases to 4.2 μm at 6 μm/s. This is due to when the growth rate is 2–6 μm/s, the higher the growth rate is, the denser the W filament is, and the smaller unit cell of area of NiAl matrix between the nanowires exposed to solution is. At the same corrosion time, the dissolution rate of NiAl matrix is slow, and the length of W wires with high growth rate is short. The length of W wires gained at 8 μm/s is 8.3–18.5 μm while their length increases to 17.9–27.5 μm at 15 μm/s. The length of W wires grown in cell growth is obviously larger than that of W wires grown in plane under the same corrosion time. This is because the area of NiAl−W eutectic alloys in the cellular growth exposed to the corrosive liquid is much larger than that of the planar ones, and the corrosion rate of NiAl matrix is faster in the former. Therefore, when the W wire is in cellular growth, the higher the growth rate, the longer and denser the wire length of W is obtained.

**Figure 4:**
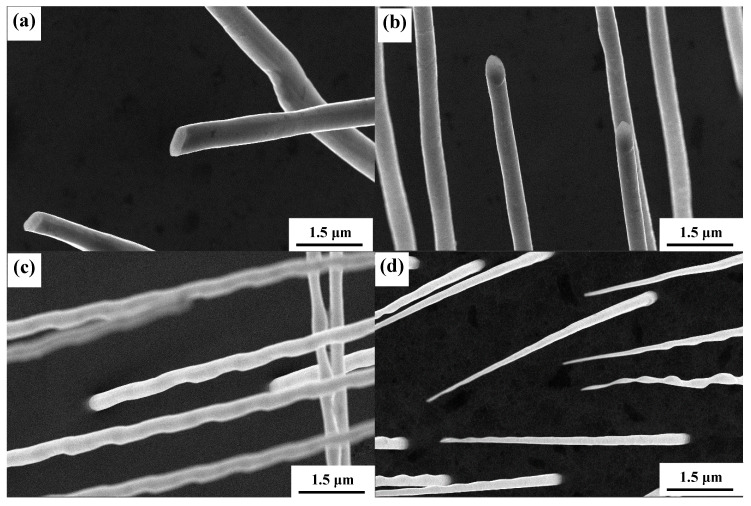
Microstructure of W fibers grown at different growth rates: (**a**) 2 μm/s, (**b**) 4 μm/s, (**c**) 8 μm/s, and (**d**) 15 μm/s. The anodization time was 1 h.

The microstructure of W wires grown at different growth rates are shown in Figure 4. The diameter of W wires decreases with increasing growth rate [24]. The diameter of W wires obtained at 2 μm/s is about 0.484 μm, and their diameter decreases to 0.331 μm at 6 μm/s. When the growth rate is 8 μm/s, the diameter of W wires is about 0.239 μm, and their diameter decreases to 0.214 μm at 15 μm/s. At a low growth rate (2–6 μm/s), the cross-section morphology of W wires presents uniformly. When at a high growth rate (8 μm/s), the W wire is not uniform, and shows ripple wave structure. The morphology of W wires even presents as needle-like at a growth rate of 15 μm/s. Formation of ripple wave shape of W wires may be due to the instability of solute enrichment and migration process. Formation of needle-like W wires is associated with the solid–liquid interface which changes from planar to cellular growth [25,26]. When at a low growth rate, the solid–liquid interface is planar growth, and the W wires are parallel growth. While at a high growth rate, the solid–liquid interface is cellular growth, and the W wires are narrow down in the cellular boundary, which results in the formation of needle-like W wires [24].

**Figure 5:**
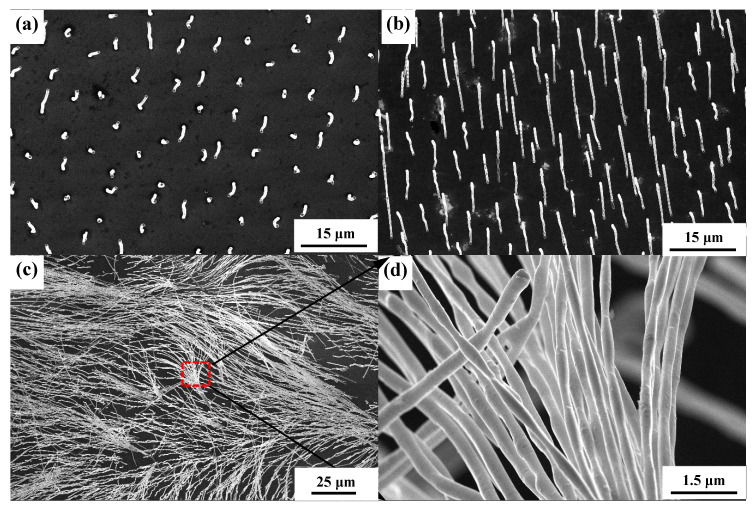
Morphology of W fibers anodized in different etching time: (**a**) 10 min, (**b**) 1 h and (**c**,**d**) 14 h.

Figure 5 shows the morphology of W wires obtained in different etching time. NiAl matrix is uniform corrosion with the increasing etching time. When the corrosion time is 10 min, the W wires with the length of 3.4–4.1 μm and diameter of 0.475–0.486 μm are exposed on the surface of NiAl matrix. When the corrosion time increases to 1 h, the length of W wire increases to 9.8–12.1 μm, and the wire diameter is 0.470–0.480 μm. The wire length increased, but the wire diameter remains almost the same. When the corrosion time is extended to 14 h, the surface of W wire is very clean without any NiAl coverage, and the length of the wire increases to 150–300 μm. Therefore, with the extension of corrosion time, the length of W wires increases, and the diameter of W wires is almost unchanged. This illustrates that the W wires are free from corrosion with the increasing of etching time, and only the NiAl matrix is uniformly passivated and dissolved.

### 3.2. Elastic Modulus of W Nanowires

**Figure 6:**
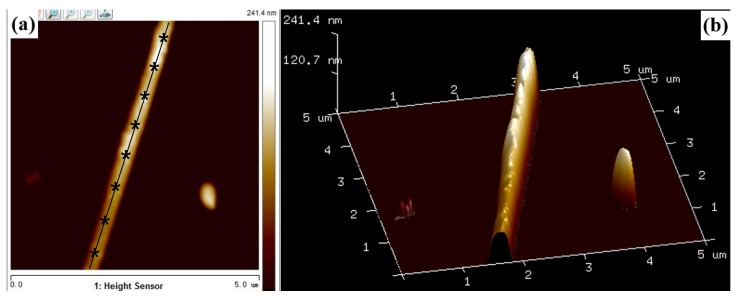
2D (**a**) and 3D (**b**) AFM images of a single W nanowire. The symbol “*” in (a) marks the measured positions of force-distance curves.

**Figure 7:**
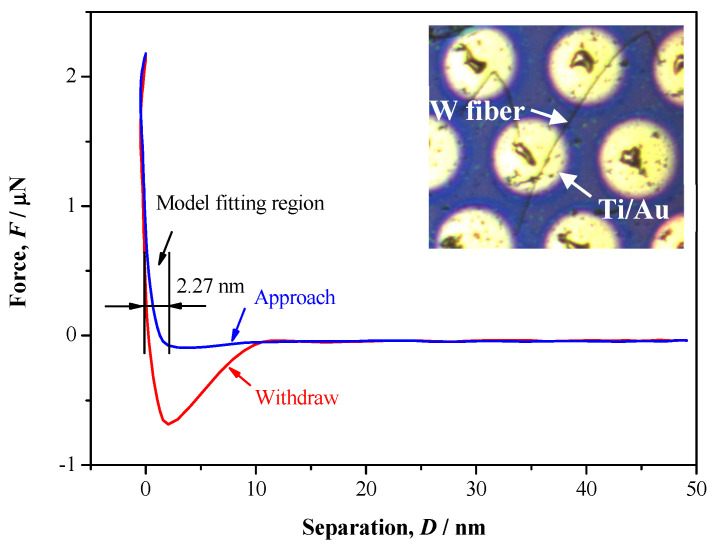
A representative *F*−*D* curve of a single metallic W nanowire measured by AFM.

**Figure 8:**
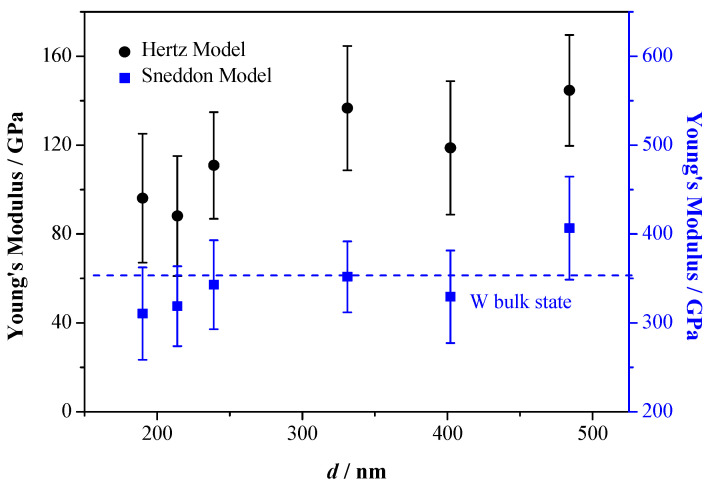
Young’s modulus of W nanofibers with different diameter fitted by Hertz and Sneddon Model.

The tapping mode of AFM is used to select a proper single W nanowire on surface of n-type Si. 2D and 3D AFM images of a single W nanowire are illustrated in Figure 6. The height of W nanowire is about 241.4 nm, and its surface roughness is about 7.35 nm. Electron diffraction [17] shows that the W nanowire is a single crystal. The peak force tapping mode of AFM is applied to measure the *F*−*D* curve of single W nanowire (Figure 7). When the AFM probe approaches or withdraws the W nanowire, it forces the W nanowire to produce deformation. There is a linear elastic deformation when the AFM probe approaches the W nanowire. The variation of linear elastic deformation with the approach force can be fit by Hertz or Sneddon model [27]. The Hertz and Sneddon models assume a linear elastic relationship between the tip and the sample, and they ignore the effects of adhesion or other surface interactions. The modulus of elasticity and Poisson’s ratio of using AFM silicon probe are 160 GPa and 0.27, respectively [28]. The Poisson’s ratio of W nanowire is 0.28 [29]. The nanoscope analysis software of AFM is used to analyze the approaching curve. Hertz and Sneddon models are used to estimate the Young’s modulus of W nanowire (Figure 8). The Young’s modulus of W nanowires based on Hertz model are 60–160 GPa, while their Young’s modulus based on Sneddon model are 260–460 GPa. Based on the cantilever beam test method by AFM, the Young’s modulus of W nanowire in Cimallar’s work [16] is 332 GPa. Gerberich adopted the nano-indentation method to test the Young’s modulus of volume W, and its value is 105–431GPa [30]. The value of Young’s modulus in W nanowire based on the Sneddon model approximates the results of Cimallar’s and Gerberich’s measurements. Therefore, the Sneddon model is more precise than Hertz model to measure the Young’s modulus of W nanowire. As illustrated in Figure 8, the Young’s modulus of W nanowire does not depend on its diameter. These results are in accord with other researchers’ studies on the relationship between the diameter of silicon nanowires and their Young’s modulus [31,32].

### 3.3. Electrical Transport Properties of W Nanowires

**Figure 9:**
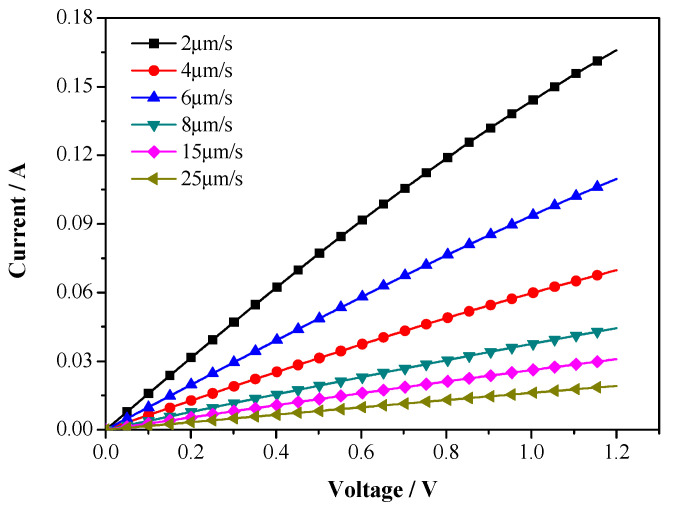
Current varies with voltage curves of W wires with different growth rate.

*I*−*V* characteristics of W nanowires in different growth rates are shown in Figure 9. The *I*−*V* characteristics of W nanowires are founded to be linear with positive slope, and this illustrates a good ohmic characteristic. The resistivity of W nanowires is calculated by the slope of *I*−*V* curve. Table 1 summarizes the diameter *d*, resistance *R*, and resistivity *ρ* of W nanowires with different growth rates *V*. As shown in Table 1, the value of *d*, *R*, and *ρ* all decrease with increasing growth rate. Decreasing in diameter with increasing growth rate has been demonstrated in directionally solidified eutectic alloy [33]. The resistivity decreases with increase of diameter can be illustrated by FS surface scattering model or MS grain boundary scattering model [19,34].

The FS model focuses on the effect of surface scattering on resistivity, and it can be described by:

(1) $$\rho_{FS}=\rho_{0}\left[1+\frac{3}{4}\left(1-P\right)\frac{\lambda }{d}\right]$$

where $\rho_{0}$ is the bulk resistivity (for tungsten $\rho_{0}=5.32\;\mu \Omega \;\mathrm{cm}$), λ is the electron mean free path in bulk (for tungsten λ=19.1 nm) [35], P (0≤P≤1) is the fraction of electrons specularly scattered at boundary, and it is often used to describe the elastic electrons on the surface reflectivity, when P=1 is a full elastic launch, when P=0 is pure diffuse scattering. d is the diameter of a wire.

The MS model gives the contribution of scattering from grain boundaries to the resistivity in cases where the dimensions of wire is greater than mean free path. The MS model can be expressed as:

(2) $$\rho_{MS}=\rho_{0}\left[1+\frac{3}{2}\left(\frac{Q}{1-Q}\right)\frac{\lambda }{g}\right]$$

where g is the mean grain size (suppose, g=d), and Q is the probability of electrons being reflected at the grain boundary, 0≤Q≤1.

Both the FS and MS model can be used to describe the effect of diameter on the resistivity of nanowires. However, a combination of FS and MS model can also be used to study the effect of diameter on the resistivity. The resistivity on different diameter in combined FS+MS model is as:

(3) $$\rho_{W}=\rho_{FS}+\rho_{MS}-\rho_{0}$$

**Figure 10:**
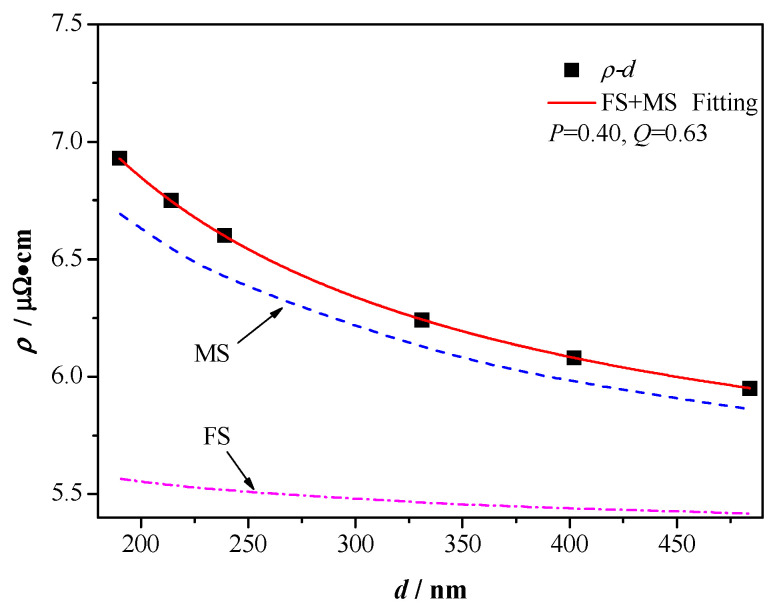
Fitting of Fuchs−Sondheimer (FS) + Mayadas−Shatzkes (MS) model on the resistivity of W wire with different diameter.

As shown in Figure 10, a single FS or MS model used to fit the resistivity on different diameter is not accurate. However, the FS+MS model fits well with the resistivity on different diameter of W nanowires (P=0.40, Q=0.63). The values of the two fitting parameters (P and Q) are similar to those previously reported (for copper nanowires, P=0.49, Q=0.64) [36]. Due to the fitting parameter, P=0.40, the electron scattering on the surface of the W wire is between full elastic scattering and diffuse scattering. Substitute the values of P and Q into the Equations (1) and (2), the value of $\rho_{MS}$ is a few times larger than $\rho_{FS}$. This illustrates that the contribution of grain boundary scattering to resistivity of W nanowires is greater than that of surface scattering.

## 4. Conclusions

The high aspect ratio tungsten nanowires have been prepared by selective etching of directionally solidified NiAl−W alloys grown at growth rates varying from 2 to 25 μm/s. By using the metallic mask template method, the Young’s modulus and resistivity of W nanowires in different diameters are analyzed. The following conclusions were drawn:

(1) W nanowires are uniform in diameter and have high aspect ratio. With increasing growth rate, the distribution of W nanowires in the NiAl matrix changes from uniform to cellular structure. The length of W nanowires increases with corrosion time, and its length can reach 300 μm at 14 h.

(2) The Young’s modulus of W nanowires based on Sneddon model are 260−460 GPa, and their values approach the bulk W. Compared with Hertz model, the Sneddon model is more accurate to estimate the Young’s modulus of W nanowires.

(3) The resistivity of W nanowires decreases with decrease of diameter. The FS + MS model fits well with the resistivity on different diameter of W nanowires.

## Figures and Tables

**Table 1:** Diameter *d*, resistance *R*, and resistivity *ρ* of W wires with different growth rates.

Growth Rate *V* (μm/s)	Diameter *d* (nm)	Resistance *R* (Ω)	Number *N*	Resistivity *ρ* (μΩ·cm)
2	484	6.48	5	5.95
4	402	15.96	3	6.08
6	331	10.36	7	6.24
8	239	24.52	6	6.60
15	214	37.56	5	6.75
25	190	61.16	4	6.93

The equation of calculating resistivity is $\rho =NR\pi d^{2}/4L$. Where *d* is the diameter of W nanowires, *R* is the resistance calculated by the slope of *I*−*V* curve, *L* is the distance between the two fingers of forked electrode, *L* = 100 μm, *N* is the number of W nanowires contacted with the two fingers of forked electrode.
